# Acquired crizotinib-resistant pulmonary adenocarcinoma and subsequent primary gallbladder cancer: A case report

**DOI:** 10.1097/MD.0000000000033162

**Published:** 2023-03-17

**Authors:** Min Zhang, Ruilin Chen, Suqun Zheng, Zhen Wang

**Affiliations:** a The First Clinical Medical College, Zhejiang Chinese Medicine University, HangZhou, China; b Department of Respiratory and Critical Care Medicine, First Affiliated Hospital of Zhejiang Chinese Medicine University, Hangzhou, China.

**Keywords:** acquired resistance, case report, crizotinib, lung adenocarcinoma, senescence

## Abstract

Proto-oncogene-oriented targeted therapy has limited benefits in elderly patients with multiple primary tumors.

**Patient concerns:**

A woman with anaplastic lymphoma kinase-positive lung adenocarcinoma developed acquired resistance after 3 years of targeted therapy with crizotinib.

**Diagnoses:**

Diagnosis of unexpected subsequent primary gallbladder tumor.

**Interventions:**

Lenvatinib was administered therapeutically. Meanwhile, next-generation sequencing results before and after crizotinib treatment were analyzed by comparing the tumor-driving mutation genes with bioinformatics methods.

**Outcomes:**

The patient died of ascites and liver failure. Furthermore, bypass activation was found to be the main reason for acquired drug resistance for this patient, and the abnormal expression of tumor suppressor genes and senescence-related genes was the likely cause of the second primary tumor.

**Lessons:**

A bioinformatic comparison of pre- and post-treatment sequencing in elderly oncology patients is of interest.

**Conclusions:**

For diagnosing, precision bioinformatics analysis and repeat biopsy are equally valuable. For therapy, potential therapy such as p53 gene replacement therapy and CAR-T therapy need to be practiced for senescence-related conditions.

**Key Points**

1. A rare case of anaplastic lymphoma kinase-resistant pulmonary adenocarcinoma with subsequent cholangiocarcinoma has been reported.
2. Bioinformatic methods were used to analyze the next-generation sequencing results of multiple samples from one such patient.
3. The impact of cellular senescence on older patients with cancer needs to be fully considered.

## 1. Introduction

Healthy aging is one of the goals of medical science. However, senescence is often associated with the deregulation of epigenetic and transcriptional control.^[1]^ Therefore, individuals aged >70 years are at an increased risk of developing multiple primary malignancies. Furthermore, targeted therapy, such as anaplastic lymphoma kinase (ALK) inhibitors, induces a complex network of secretory signals, promoting the survival of cancer cells sensitive to the drug.^[2]^ A study based on Surveillance, Epidemiology, and End Results database showed that lung cancer patients with a duration >120 months had an increased risk of secondary primary gallbladder cancer.^[3]^ Owing to tumor heterogeneity, there may be multiple mechanisms of treatment resistance and the development of multiple primary tumors in the same patient. Here we present a typical case in accordance with the CARE Guidelines. Written informed consent from the patient was obtained for the publication of the case details and any accompanying images.

## 2. Case presentation

**Figure 1.**
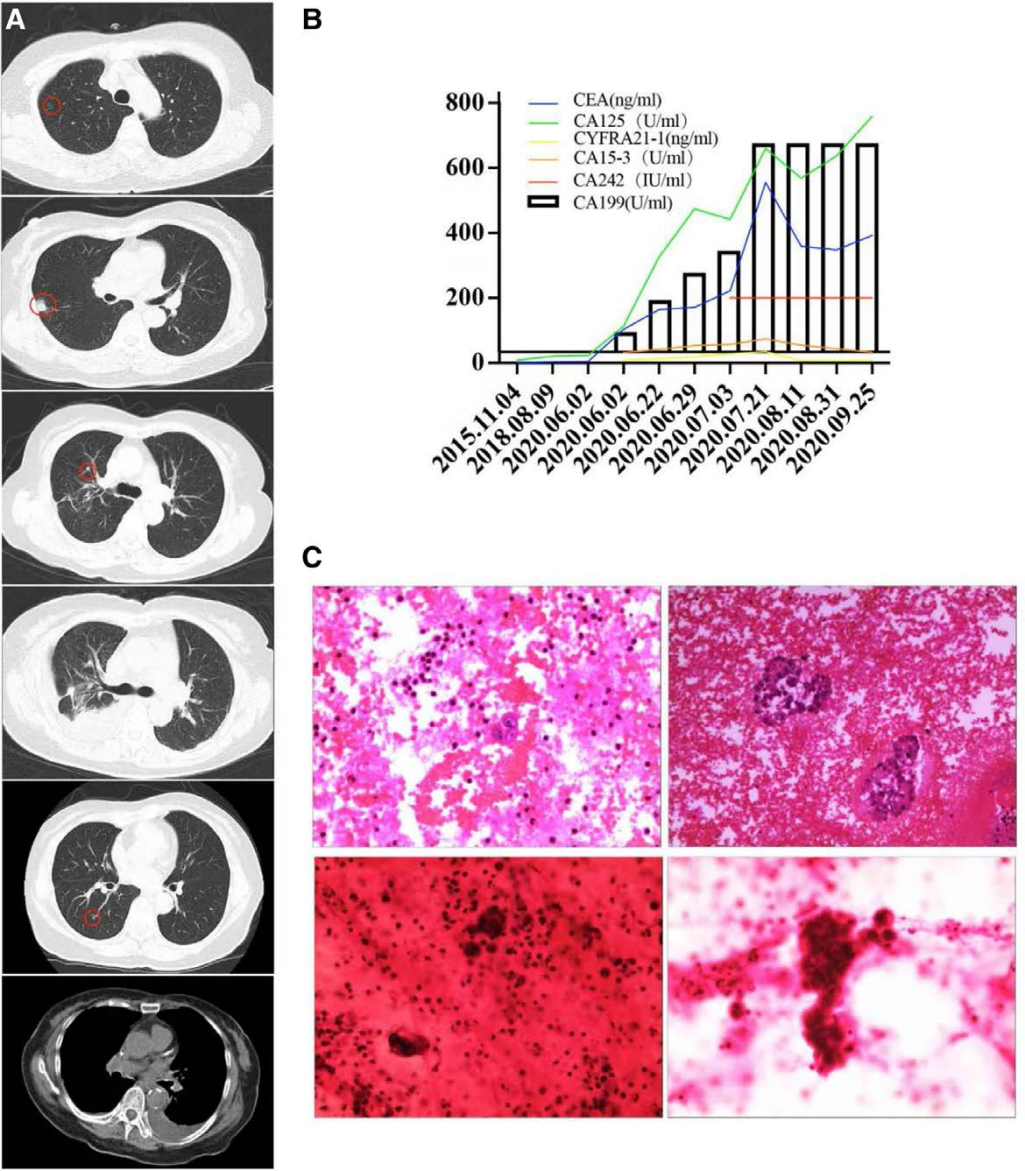
(A) The chest CT outcomes during the whole treatment. Ground-glass nodules in the upper lobe of the right lung (diameter 5.7 mm) and subpleural nodules in the upper lobe of the right lung (diameter 12.8 mm) were first detected by chest CT on July 15, 2015. A right upper lobe nodule (approximate diameter 7.9 mm) with a short marginal burr was observed 9 months after surgery. Pleural effusion and pericardial effusion were detected on July 1, 2017. The 7-mm diameter ground glass nodules were observed 24 months after initiation of crizotinib treatment. There was a recurrence of pleural effusion and pericardial effusion on October 8, 2020. (B) The trend of change in serum cancer markers. (C) Adenocarcinoma cells were found in pericardial effusion and pericardial effusion wax blocks on June 15, 2017. Adenocarcinoma cells were found in bile drainage smears on July 7, 2020. CT = computed tomography.

An 85-year-old Asian woman, a retired gynecologist, presented with a fever for 3 days. Five years back she was diagnosed with lung adenocarcinoma (Fig. 1A) and underwent wedge resection of the right lung malignant tumor (initial tumor stage: T2aN1M0 [Union for International Cancer Control], IIB [American Joint Committee on Cancer], histology: low-grade adenocarcinoma G3). Immunohistochemistry results were: TTF-1(+), CK7(+), Ki-67 positivity of 50% (Fig. 1C). Three years ago, she was started on crizotinib 200 mg twice daily because of the detection of ALK gene rearrangements in pericardial and pleural effusion. She showed tolerable side effects such as rash and diarrhea. Her past medical history revealed first-degree atrial ventricular block, allergy to penicillin, and compression fracture of the lumbar vertebra. She denied any history of alcohol or tobacco intake. There was no family history of cancer. On arrival, she was febrile (body temperature 99.86°F), and had bradycardia (heart rate 54 beats/min), and hypotension (blood pressure 111/43 mm Hg). Laboratory tests showed markedly elevated serum levels of CA19-9 (1125.3 U/mL; reference range, 0–37 U/mL) and CEA (103.1 ng/mL; reference range, 0–5 ng/mL) (Fig. 1B). Positron emission tomography/computed tomography showed no obvious signs of tumor recurrence in the operation area, while the metabolism of fluorodeoxyglucose was enhanced in the duodenal papilla area, gallbladder wall, left lobe of the liver, and retroperitoneal lymph nodes (standardized uptake value max = 7.6). After multi-disciplinary consultation, she was subjected to ultrasound-guided percutaneous catheterization and drainage of the gallbladder. The results of immunohistochemistry were: CK7(+), TTF-1(−), CKpan(+), CK19(−), CEA(+), and Ki-67(−). Both blood and bile samples were subjected to next-generation sequencing. The results showed tumor-specific mutations such as missense mutation in exon 9 of PIK3CA P. e542k and germline genes mutations of PRF1, SBDS, and a tumor mutation burden of 12.89 mutations/Mb (Table 1). Based on the above information, primary rather than metastatic biliary tract tumors were considered. Therefore, lenvatinib and supportive treatments were administered during hospitalization; unfortunately, she died due to ascites and liver failure.

**Table 1:** Variants identified in the post-ALK TKI biopsy sample.

	cDNA change	AA change
PIK3CA	c.1624G > A	p.E542K
NFE2L2	c.88C > T	p.L30F
APC	c.7508G > A	p.G2503E
GRBB4	c.308G > A	p.R103H
GRM8	c.2152C > T	p.P718S
KNX2-1	c.770G > A	p.R257H
PKHD1	c.8630C > T	p.S2877L
TSC1	c.2111A > G	p.Y704C
TP53	c.835G > T	p.G279W
TP53	c.833C > T	p.P278L
PRF1	c.1090_109delCT	p.L264Efs*93
SBDS	c.s58 + 2T > C	–
ERCC1	c.354T > C	p.N118=
SGTT1	–	–
MTHFR	c.665C > T	p.A222V
NQO1	c.559C > T	p.P187S
TYMS	c.-97_70CCGCGCCACTTGGCCTGCCTCCGTCCCG[3]	–
TYMS	c.*450_*455delAAGTTA	–

ALK = anaplastic lymphoma kinase, TKI = tyrosine kinase inhibitor.

**Figure 2.**
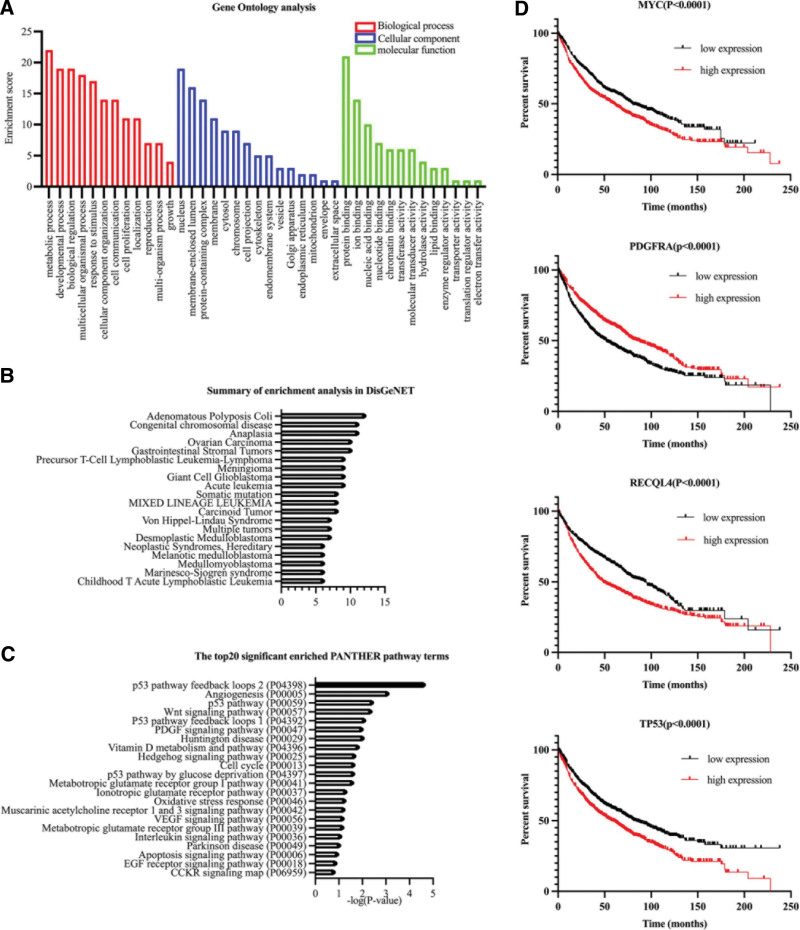
(A) Results of Gene Ontology (GO) analysis (WebGestalt platform) sorted according to the enrichment score. (B) The enrichment analysis related to the disease was performed using the Metascape website, and the annotation data were obtained from the DisGeNet database. (C) Enrichment analysis of the pathway was completed through the PANTHER database; Fisher exact test was used and corrected by false discovery rate, and the results were sorted according to the –log (*P* value). (D) Survival analysis was performed using the Kaplan–Meier Plotter database, with overall survival as the key event, and Cox regression was used for statistics.

Bioinformatics analyses were performed to unravel the mechanism of acquired drug resistance and the development of multiple primary tumors. Sequenced samples were collected from blood, pleural effusion, and pericardial effusion samples collected in 2017, and blood, bile drainage fluid supernatant, and the precipitate collected in 2020. Potential cancer-driving regulatory mutations were prioritized using the Varnote-CAN online tool (http://mulinlab.tmu.edu.cn/gwas4d), and 23 potential driving mutant genes were obtained.^[4]^ The results of the Gene Ontology analysis suggested that the key driving mutant genes were mainly concentrated in the nucleus, membrane-enclosed lumen, and protein-containing complex. The biological processes involved were mainly the metabolic process, developmental process, and biological regulation. Molecular functions included protein binding and ion binding, nucleic acid binding, etc (Fig. 2A).^[5]^ Disease-related gene enrichment analysis indicated that the driver genes were not only closely related to congenital diseases (such as congenital chromosomal diseases) but also inseparable from diseases such as ovarian carcinoma, gastrointestinal stromal tumors, and multiple other tumors (Fig. 2B).^[6,7]^ Pathway analysis revealed the dysfunction of the p53 pathway feedback loops 2 and angiogenesis pathways (Fig. 2C).^[8]^ Survival analysis showed that abnormal expression of 16 out of the 23 genes significantly affected the prognosis of lung cancer,^[9]^ including WT1, CDH4, CYLD, GRIN2A, and other tumor suppressor genes.^[10]^

Of all the single nucleotide mutations, the transition from cytosine to thymine (C > T) was the most common, a feature associated with cytosine deamination associated with aging.^[11]^ MYC, PDGFRA, RECQL4, and TP53 are all classic senescence-associated genes and have been shown to influence the overall survival of patients with lung cancer (Fig. 2D).^[12]^

## 3. Discussion

### 3.1. Aberrant tumor suppressor genes are responsible for ALK-acquired resistance in this patient

Crizotinib (Xalkori, PF-02341066), the first-generation ALK inhibitor, has become the standard first-line therapy for ALK-positive non-small cell lung cancer allowing survivals up to 5 years.^[13,14]^ According to a study, 6.7 to 18.2% of patients acquire resistance within 12 months.^[15]^ The presence of drug resistance in this patient was not surprising given that the patient was receiving the medication for the last 36 months.

The mechanisms of resistance to ALK inhibitors can be divided into 2 categories: ALK-dependent and ALK-independent processes.^[16]^ The first mechanism includes ALK secondary mutations, while the second mechanism includes bypass track mechanisms, such as PIC3CA and TP53 mutations, as observed in this patient.^[17,18]^

While crizotinib remained effective in this patient, the “Pandora’s box” of aberrant tumor suppressor genes was turned on. Reports have suggested that aberrations of RB and TP53 are involved in the rare transition of lung adenocarcinoma to SCLC.^[19,20]^ The abnormality of tumor suppressor genes leads to dyshomeostasis of cell cycle control, promoting the survival of tumor cells and improving the possibility of multiple primary tumors. Although the gallbladder is rarely a metastatic site for malignancy,^[21]^ and subsequent primary gallbladder cancers also rarely occur in patients with lung adenocarcinoma,^[22]^ new lesions still developed. Under such conditions, new therapeutic approaches such as p53 gene replacement therapy and PIK3CA inhibitors may have been appropriate for this patient but regrettably, these are still under investigation.

### 3.2. Dual effects of cellular senescence on tumor progression

Aging is an inevitable phenomenon that is often accompanied by tumorigenesis. Approximately 10% of individuals over the age of 65 years show clonal expansion of cancer-associated mutations in their blood.^[23]^ A Chinese lung cancer gene mutation study showed that ALK, KRAS, and BRAF gene abnormalities were mainly found in patients aged <60 years, while other genes were dominant in elderly patients.^[24]^ Therefore, proto-oncogene is not the only important reference factor for elderly cancer patients, and the corresponding targeted therapies such as lenvatinib mentioned above, have limited efficacy.

On the other hand, aging is a double-edged sword for elderly cancer patients. Cellular senescence can curtail the development of cancer by inhibiting proliferation, thereby promoting early survival; however, it ultimately limits longevity due to the accumulation of dysfunctional senescent cells and the proliferation of inactive stem cells.^[25]^

Furthermore, it is not difficult to explain why multiple primary tumors occurred in this patient. DNA methylation and stem cell division during aging contribute to tumor formation and are risk factors for heterogeneous second primary tumors.^[26]^ From this perspective, treatment strategies targeting cellular senescence, such as senolytic CAR T cells therapy, have broad application prospects in elderly cancer patients,^[27,28]^ especially those with acquired resistance or multiple primary tumors.^[29,30]^

## 4. Conclusion

We report a case of typical ALK mutated lung adenocarcinoma with acquired resistance who developed an atypical second primary tumor. Enrichment analysis of tumor-associated mutated driver genes was performed by bioinformatics analysis of the patient’s next-generation sequencing results. The findings suggest that the impact of cellular senescence in older patients with cancer needs to be fully considered. The possibility of multiple primary tumors needs to be evaluated after the development of resistance to ALK-tyrosine kinase inhibitor, just like histological transformation. Precision bioinformatics analysis and repeat biopsy are equally valuable. Further studies are required to explore the treatment modalities for older cancer patients with more complex mechanisms of driver mutations. Senescence-targeted therapy holds promise.

## Author contributions

**Conceptualization:** Suqun Zheng, Zhen Wang.

**Formal analysis:** Ruilin Chen.

**Project administration:** Zhen Wang.

**Writing – original draft:** Min Zhang.

**Writing – review & editing:** Min Zhang.
